# Bmi-1 promotes invasion and metastasis, and its elevated expression is correlated with an advanced stage of breast cancer

**DOI:** 10.1186/1476-4598-10-10

**Published:** 2011-01-28

**Authors:** Bao-Hong Guo, Yan Feng, Rong Zhang, Li-Hua Xu, Man-Zhi Li, Hsiang-Fu Kung, Li-Bing Song, Mu-Sheng Zeng

**Affiliations:** 1 State Key Laboratory of Oncology in South China and Department of Experimental Research, Sun Yat-Sen University Cancer Center, Guangzhou, PR China; 2 Stanley Ho Centre for Emerging Infectious Diseases, School of Public Health and Primary Care, Prince of Wales Hospital, the Chinese University of Hong Kong, Shatin, N.T., Hong Kong, PR China; 3 Department of Endoscopy and Laser treatment, Sun Yat-Sen University Cancer Center, Guangzhou, PR China

## Abstract

**Background:**

B-lymphoma Moloney murine leukemia virus insertion region-1 (Bmi-1) acts as an oncogene in various tumors, and its overexpression correlates with a poor outcome in several human cancers. Ectopic expression of Bmi-1 can induce epithelial-mesenchymal transition (EMT) and enhance the motility and invasiveness of human nasopharyngeal epithelial cells (NPECs), whereas silencing endogenous Bmi-1 expression can reverse EMT and reduce the metastatic potential of nasopharyngeal cancer cells (NPCs). Mouse xenograft studies indicate that coexpression of Bmi-1 and H-Ras in breast cancer cells can induce an aggressive and metastatic phenotype with an unusual occurrence of brain metastasis; although, Bmi-1 overexpression did not result in oncogenic transformation of MCF-10A cells. However, the underlying molecular mechanism of Bmi-1-mediated progression and the metastasis of breast cancer are not fully elucidated at this time.

**Results:**

Bmi-1 expression is more pronouncedly increased in primary cancer tissues compared to matched adjacent non-cancerous tissues. High Bmi-1 expression is correlated with advanced clinicopathologic classifications (T, N, and M) and clinical stages. Furthermore, a high level of Bmi-1 indicates an unfavorable overall survival and serves as a high risk marker for breast cancer. In addition, inverse transcriptional expression levels of Bmi-1 and E-cadherin are detected between the primary cancer tissues and the matched adjacent non-cancerous tissues. Higher Bmi-1 levels are found in the cancer tissue, whereas the paired adjacent non-cancer tissue shows higher E-cadherin levels. Overexpression of Bmi-1 increases the motility and invasive properties of immortalized human mammary epithelial cells, which is concurrent with the increased expression of mesenchymal markers, the decreased expression of epithelial markers, the stabilization of Snail and the dysregulation of the Akt/GSK3β pathway. Consistent with these observations, the repression of Bmi-1 in highly metastatic breast cancer cells remarkably reduces cellular motility, invasion and transformation, as well as tumorigenesis and lung metastases in nude mice. In addition, the repression of Bmi-1 reverses the expression of EMT markers and inhibits the Akt/GSK3β/Snail pathway.

**Conclusions:**

This study demonstrates that Bmi-1 promotes the invasion and metastasis of human breast cancer and predicts poor survival.

## Background

The processes of invasion and metastasis that cause mortality in patients are extraordinarily distinctive features of breast cancer progression [1]. Although lymph-node metastasis, large tumor size, and poorly-differentiated histopathological grade are commonly considered to be established prognostic markers related to metastasis [2], distant metastasis still occurs in 20-30% of the patients with negative lymph-node involvement [3]. Thus far, Human Epidermal Growth Factor Receptor 2 (HER-2/neu) [4], c-myc [5] and HOXB9 [6] have emerged as predictors of the risk of metastasis in breast cancer. The aberrant expression of these factors may induce the expression of growth and angiogenic factors in tumors, leading to increased local concentrations of these factors within the tumor microenvironment and thus favoring tumor progression [6]. Recently, a new genomic test (gene-expression profiling) has been suggested to predict the clinical outcome more accurately than the traditional clinical and pathological standards [7,8]. However, it is an open question as to whether this method will enter into the clinical routine for staging and grading [9]. Although these new markers and methods have been implicated, the molecular mechanism of breast cancer metastasis remains far from being fully understood due to the heterogeneity of this cancer and represents a new prerequisite for developing better treatment strategies.

The polycomb (PcG) proteins constitute a global system with important roles in multi-cellular development, stem cell biology and cancer [10]. B-lymphoma Moloney murine leukemia virus insertion region-1 (Bmi-1), a member of the PcG family of transcription repressors, has emerged as a Myc-cooperating oncogene in murine lymphomas [11,12]. Bmi-1 can not only lead human mammary epithelial cells (HMECs) to bypass senescence and immortalize, but it also can play a key role in human breast cancer [13,14]. Moreover, a significant correlation has been observed between Bmi-1 expression and axillary lymph node metastasis in invasive ductal breast cancer [15]. These findings suggest that Bmi-1 could be involved in the carcinogenesis and metastasis of breast cancer. Although increasing evidence has shown that Bmi-1 expression is associated with unfavorable prognosis [16,17], other studies have not confirmed these findings [18,19]. Bmi-1 protein is detected in only 25% of African breast cancer patients and is associated with a low histological grade [18]. Additionally, higher Bmi-1 mRNA expression has been observed in early-stage patients without lymph node metastasis [20]. In contrast, up-regulation of Bmi-1 was shown to be associated with the invasion of nasopharyngeal carcinomas and to predict poor survival [21]. In colon cancer and gastric cancer, Bmi-1 expression is significantly correlated with nodal involvement, distant metastasis and clinical stage [22-24]. Furthermore, metastatic melanoma tissues and cell lines show much higher expression of Bmi-1 than primary melanoma tissues and cell lines [25]. Furthermore, knockdown of Bmi-1 contributes to decreased invasiveness of cervical cancer cells and gastric cells [4,26]. These findings indicate that Bmi-1 contributes to increased aggressive behavior of cancer cells. Bmi-1 overexpression can promote epithelial-mesenchymal transition (EMT) in NPECs, whereas Bmi-1 knockdown can reverse EMT and reduce the metastasis of nasopharyngeal carcinoma cells (NPC) [27]. Although Bmi-1 overexpression alone did not result in oncogenic transformation of MCF-10A cells, overexpression of Bmi-1 together with H-Ras did induce an aggressive and metastatic phenotype, with the unusual occurrence of breast cancer brain metastasis [28]. In spite of the aforementioned link between Bmi-1 and cancer, very few studies have focused on the molecular mechanism and clinical outcome of Bmi-1 in breast cancer metastasis.

The metastasis of cancer is a complex and multi-step process, including a series of successive and dynamic events along with alterations to cell morphology and biological function [29]. After acquiring the ability to undergo EMT, cancers are prone to metastasize and establish secondary tumors at distant sites [30,31]. During EMT, epithelial cells acquire mesenchymal-like properties, which increase cell motility, and lose epithelial-like properties, which decrease intercellular adhesion [32,33]. Loss of E-cadherin is a hallmark of the invasive phase of cancer, and E-cadherin can be repressed by certain dominant transcriptional factors, such as Snail, ZEB, Twist, and basic Helix-Loop-Helix family proteins (bHLH) [34-36]. Snail-induced EMT is an important breakthrough in the study of metastasis, providing new insights into the molecular mechanisms of tumor invasion [37,38]. Moreover, Snail expression is associated with E-cadherin repression and metastasis in breast cancer cells, as well as in other cancer cell types [39-43]. In addition to Snail, numerous agents are involved in breast cancer EMT, such as components of the Six1, YB-1 and miRNA-200 families [44-46]. Therefore, it is important to understand whether Bmi-1 can regulate EMT during breast cancer progression and metastasis.

The present study focuses on the expression patterns and roles of Bmi-1 in breast cancer tissues and cells to investigate the involvement of Bmi-1 in breast cancer metastasis. We demonstrate that Bmi-1 not only is increased in breast cancer tissues compared with adjacent non-cancerous tissues but also is associated with clinical features, such as tumor size, lymph node involvement, distant metastasis and clinical stage. High Bmi-1 expression predicts an unfavorable patient prognosis and serves as a high risk indicator in breast cancer. Furthermore, we also shed light on the biological impact of Bmi-1 on the invasive and metastatic properties of breast cancer cells. The overexpression of Bmi-1 enhances the motility and invasiveness of immortalized HMECs, facilitates concurrent EMT-like molecular changes, and promotes the stabilization of Snail and the activation of the Akt/GSK3β pathway. Consistent with these observations, repression of Bmi-1 in highly metastatic breast cancer cells markedly reduces cell motility and invasion, as well as tumorigenesis and lung metastases in nude mice. In addition, repression of Bmi-1 reverses the expression of EMT markers and inhibits the Akt/GSK3β pathway. Taken together, these results provide evidence that breast cancers expressing Bmi-1 exhibit aggressive and metastatic properties.

## Results

### Increased expression of Bmi-1 in breast cancer tissues

**Figure 1:**
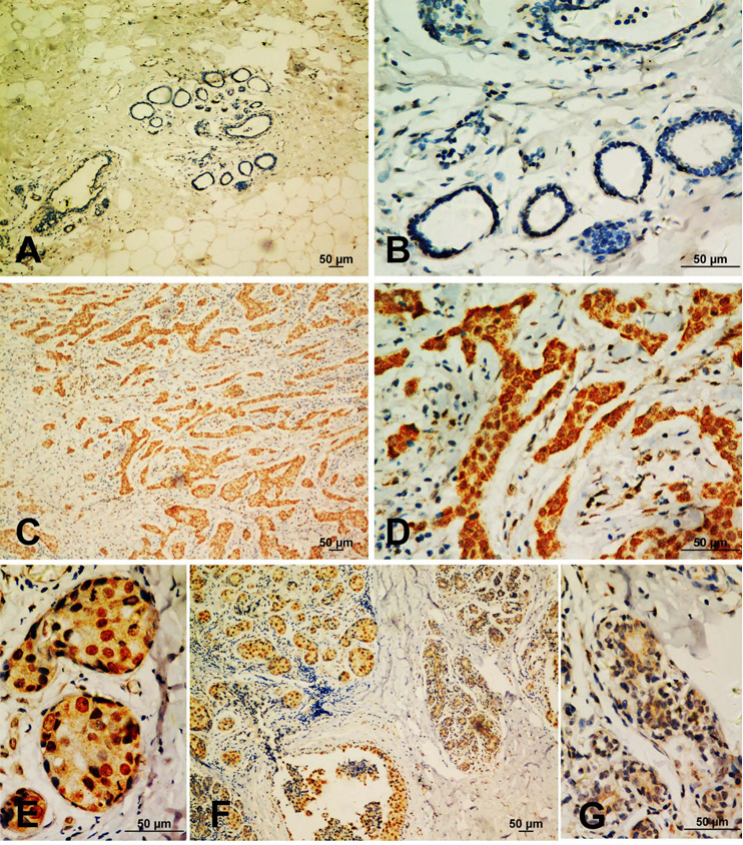
**Increased expression of Bmi-1 in breast cancer tissues**. **(A, B) **Bmi-1 showed no or weak staining in the adjacent non-cancerous tissue. **(C, D) **Strong Bmi-1 staining was detected in the primary breast cancer tissue. **(E, F, J) **Cancer showed high Bmi-1 expression and nuclear staining, whereas the adjacent mammary gland lobule showed low expression and cytoplasmic staining (100X and 400X).

To reveal the role of Bmi-1 in breast cancer, immunohistochemistry was performed to measure Bmi-1 expression in breast cancer tissues and adjacent non-cancerous tissues. Table 1 presents the percentage of positive cells and staining intensity of Bmi-1 expression in relation to clinicopathologic features. Bmi-1 expression was significantly increased in primary cancer tissues compared with matched adjacent non-cancerous tissues (χ^2 ^*= *20.237, ***P < 0.001, Table 2). Only 35.9% (14 of 39) of matched adjacent non-cancerous tissues displayed high expression of Bmi-1, and the remaining tissues (64.1%, 25 of 39) were scored as having no or low expression of Bmi-1(Figure 1 A, B). However, as many as 72.2% (182 of 252) of the cancer tissues were defined as manifesting high Bmi-1 expression (Figure 1 C, D). Positive staining was observed in 96.5% (241) of the cases. It was noted that more intense staining was observed in cancer tissues than the adjacent hyperplastic lobular glandules (Figure 1 E, F, G). Interestingly, Bmi-1 could be detected in both the nuclei and cytoplasm in the adjacent non-cancer cells but was mainly localized to the nuclei of cancer cells (Figure 1 E, F, G). Among the adjacent non-cancerous tissues, no Bmi-1 signal was detected by staining in 30.8% (12 of 39) of the samples. Only 28.2% (11 of 39) of the samples showed nuclear staining, and the remaining 41.0% (16 of 39) of the samples exhibited cytoplasmic staining. Of the cancer tissues, however, 75.4% (190 of 252) of the samples were stained in the nucleus and 24.6% (62 of 252) in the cytoplasm. These results indicate that Bmi-1 protein seems to be localized in the nucleus of the majority of breast cancer cells and in the cytoplasm of most non-cancer cells.

**Table 1:** The percentage of positive cells and staining intensity of Bmi-1 expression related to clinicopathologic features

	Percentage of Positive Cells				Staining Intensity			
		
	1	2	3	4	1	2	3	4
	N	N	N	N	N	N	N	N
	(%)	(%)	(%)	(%)	(%)	(%)	(%)	(%)
Age								
≤45 year	11	38	20	30	5	33	35	26
	(11.1)	(38.4)	(20.2)	(30.3)	(5.1)	(33.3)	(35.4)	(26.3)
>45 year	16	40	21	64	5	46	54	36
	(11.3)	(28.4)	(14.9)	(45.4)	(3.5)	(32.6)	(38.3)	(25.5)
T Classification								
T1	8	21	0	16	5	23	8	9
	(17.8)	(46.7)	(0.0)	(35.6)	(11.1)	(51.1)	(17.8)	(20.0)
T2	16	36	23	60	4	45	52	34
	(11.9)	(26.7)	(17.0)	(44.4)	(3.0)	(33.3)	(38.5)	(25.2)
T3	4	16	14	18	2	9	26	15
	(7.7)	(30.8)	(26.9)	(34.6)	(3.8)	(17.3)	(50.0)	(28.8)
T4	0	7	5	8	0	5	9	6
	(0.0)	(35.0)	(25.0)	(40.0)	(0.0)	(25.0)	(45.0)	(30.0)
N Classification								
N0	18	35	6	31	6	44	24	16
	(20.0)	(38.9)	(6.7)	(34.4)	(6.7)	(48.9)	(26.7)	(17.8)
N1	10	35	28	59	5	30	57	40
	(7.6)	(26.5)	(21.2)	(44.7)	(3.8)	(22.7)	(43.2)	(30.3)
N2	0	6	6	10	0	5	10	7
	(0.0)	(27.3)	(27.3)	(45.5)	(0.0)	(22.7)	(45.5)	(31.8)
N3	0	4	2	2	0	3	4	1
	(0.0)	(50.0)	(25.0)	(25.0)	(0.0)	(37.5)	(50.0)	(12.5)
M Classification								
M0	27	67	30	83	10	72	79	46
	(13.0)	(32.4)	(14.5)	(40.1)	(4.8)	(34.8)	(38.2)	(22.2)
M1	1	13	12	19	1	10	16	18
	(2.2)	(28.9)	(26.7)	(42.2)	(2.2)	(22.2)	(35.6)	(40.0)
Clinical Stage								
I	3	14	0	6	2	16	2	3
	(13.0)	(60.9)	(0.0)	(26.1)	(8.7)	(69.6)	(8.7)	(13.0)
II	21	33	6	34	7	41	30	16
	(22.3)	(35.1)	(6.4)	(36.2)	(7.4)	(43.6)	(31.9)	(17.0)
III	3	20	24	43	1	15	47	17
	(3.3)	(22.2)	(26.7)	(47.8)	(1.1)	(16.7)	(52.2)	(30.0)
IV	1	13	12	19	1	10	16	18
	(2.2)	(28.9)	(26.7)	(42.2)	(2.2)	(22.2)	(35.6)	(40.0)
ER Presence								
Negative	19	37	23	42	8	39	42	32
	(15.7)	(30.6)	(19.0)	(32.7)	(6.6)	(32.2)	(34.7)	(26.4)
Positive	9	43	19	60	3	43	53	32
	(6.9)	(32.8)	(14.5)	(45.8)	(2.3)	(32.8)	(40.5)	(24.4)
PR Presence								
Negative	16	30	23	38	5	33	40	29
	(15.0)	(28.0)	(21.5)	(35.5)	(4.7)	(30.8)	(37.4)	(27.1)
Positive	12	50	19	64	6	49	55	35
	(8.3)	(34.5)	(13.1)	(44.1)	(4.1)	(33.8)	(37.9)	(24.1)
HER-2 Presence								
Negative	9	12	8	22	4	17	14	16
	(17.6)	(23.5)	(15.7)	(43.1)	(7.8)	(3.3)	(27.5)	(31.4)
Positive	12	30	16	50	5	34	38	31
	(11.1)	(27.8)	(14.8)	(46.3)	(4.6)	(31.5)	(35.2)	(28.7)

Four categories of the percentage of positive cells: 1: ≤5%; 2: 6%-35%; 3: 36%-70%; 4: ≥71% (40X). Four categories of the staining intensity: 1: negative; 2: weak; 3: moderate; 4: strong.

**Table 2:** Difference of Bmi-1 expression between breast cancer tissues and adjacent non-cancerous tissues

	Bmi-1 Expression		***χ***^***2***^	*P-value*
			
	Low	High		
	N (%)	N (%)		
Tissues			20.237	*<0.001*
Non-cancer	25 (64.1)	14 (35.9)		
Cancer	70 (27.8)	182 (72.2)		

Four categories of the percentage of positive cells: 1: ≤5%; 2: 6%-35%; 3: 36%-70%; 4: ≥71% (40X). Four categories of the staining intensity: 1: negative; 2: weak; 3: moderate; 4: strong.

Final score = score of percentage of positive cells × score of staining intensity. If the final score was >4, Bmi-1 expression was considered high, otherwise, Bmi-1 expression was considered low.

### Correlation between Bmi-1 expression and clinical aggressiveness of breast cancer

We further examined possible correlations between Bmi-1 expression profiles and the patients' clinicopathologic characteristics. As presented in Table 3, our analysis of 252 primary breast cancer cases revealed that Bmi-1 expression was strongly correlated with larger tumor size (P < ***0.001), lymph node involvement (P < ***0.001), distant metastasis (P < ***0.001) and advanced clinical stage (P < ***0.001). These observations suggested a correlation between increased Bmi-1 expression and clinical progression in breast cancer. However, no evident correlations were observed between Bmi-1 expression profiles and other clinical/laboratory features, including age, estrogen receptor (ER), progesterone receptor (PR) and HER-2.

**Table 3:** Correlation between Bmi-1 expression and the clinicopathologic features of breast cancer

	Bmi-1 Expression		Total	***χ***^***2***^	*P-value*
				
	Low	High			
	N (%)	N (%)	N (%)		
Age				0.36	*0.551*
≤45 year	26 (26.3)	73 (73.7)	99 (41.2)		
>45 year	42 (29.8)	99 (70.2)	141 (58.8)		
T Classification				30.92	*<0.001*
T1	26 (57.8)	19 (42.2)	45 (17.9)		
T2	35 (25.9)	100 (74.1)	135 (53.6)		
T3	9 (17.3)	43 (82.7)	52 (20.6)		
T4	0 (0.0)	20 (100.0)	20 (7.9)		
N Classification				46.45	*<0.001*
N0	48 (53.3)	42 (46.7)	90 (35.7)		
N1	20 (15.2)	112 (84.8)	132 (52.4)		
N2	0 (0.0)	22 (100.0)	22 (8.7)		
N3	2 (25.0)	6 (75.0)	8 (3.2)		
M Classification				14.87	*<0.001*
M0	68 (32.9)	139 (67.1)	207 (82.7)		
M1	2 (4.4)	43 (95.6)	45 (17.9)		
Clinical Stage				82.06	*<0.001*
I	16 (69.6)	7 (30.4)	23 (9.1)		
II	48 (51.1)	46 (48.9)	94 (37.3)		
III	4 (4.4)	86 (95.6)	90 (35.7)		
IV	2 (4.4)	43 (95.6)	45 (17.9)		
ER Presence				1.53	*0.217*
Negative	38 (31.4)	83 (68.6)	121 (48.0)		
Positive	32 (24.4)	99 (75.6)	131 (52.0)		
PR Presence				0.04	*0.837*
Negative	29 (27.1)	78 (72.9)	107 (42.5)		
Positive	41 (28.3)	104 (71.7)	145 (57.5)		
HER-2 Presence				0.71	*0.400*
Negative	17 (33.3)	34 (66.7)	51 (32.1)		
Positive	29 (26.9)	79 (73.1)	108 (67.9)		

Four categories of the percentage of positive cells: 1: ≤5%; 2: 6%-35%; 3: 36%-70%; 4: ≥71% (40X).

Four categories of the staining intensity: 1: negative; 2: weak; 3: moderate; 4: strong.

Final score = score of percentage of positive cells × score of staining intensity. If the final score was >4, Bmi-1 expression was considered high, otherwise, Bmi-1 expression was considered low.

### High Bmi-1 expression is associated with an unfavorable prognosis

The characteristics of breast cancer patients relevant to overall survival are shown in Additional file 1, table S1. As expected, the clinicopathologic classification (T, N, M) and clinical stage were important prognostic indicators in breast cancer (P < ***0.001, respectively). The presence of PR also appeared to have a clinical prognostic value (P = **0.002), but age and expression of ER or HER-2 did not. The overall survival (OS) was 97.1% (232 of 239) after the first year of follow-up, 86.6% (207 of 239) after the second year, 77.0% (184 of 239) after the third year, 71.1% (170 of 239) after the fourth year and 49.4% (118 of 239) after the fifth year.

**Figure 2:**
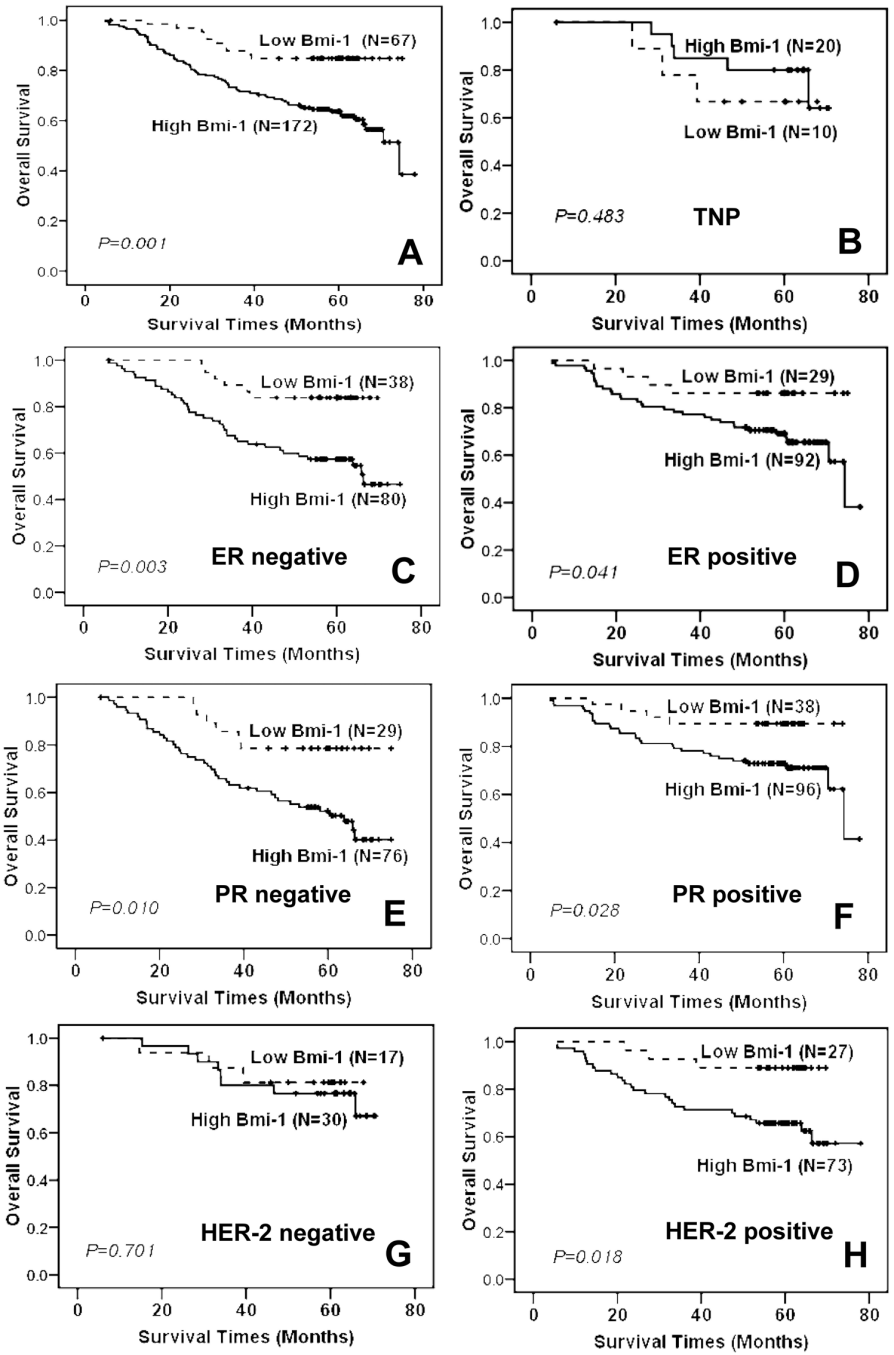
**Influence of Bmi-1 expression on the overall survival**. **(A)**The cumulative overall survival exhibited a significant difference based on Bmi-1 expression, assessed by Kaplan-Meier curves in primary breast cancer tissues (***P = 0.001). **(B) **No difference in the overall survival curve was observed according to Bmi-1 expression in the triple negative phenotype (TNP, ER^-^, PR^-^, and HER-2^-^) subgroup (*P *= 0.483). **(C, D, E, F) **High Bmi-1 expression was correlated with unfavorable prognosis irrelevant of the presence of ER (**P = 0.003, *P = 0.041) or PR (**P = 0.010, *P = 0.028). **(G, H) **The survival curves were significantly different according to Bmi-1 expression in the HER-2 positive panel (*P = 0.018), but not in HER-2 negative panel (*P *= 0.701).

**Figure 3:**
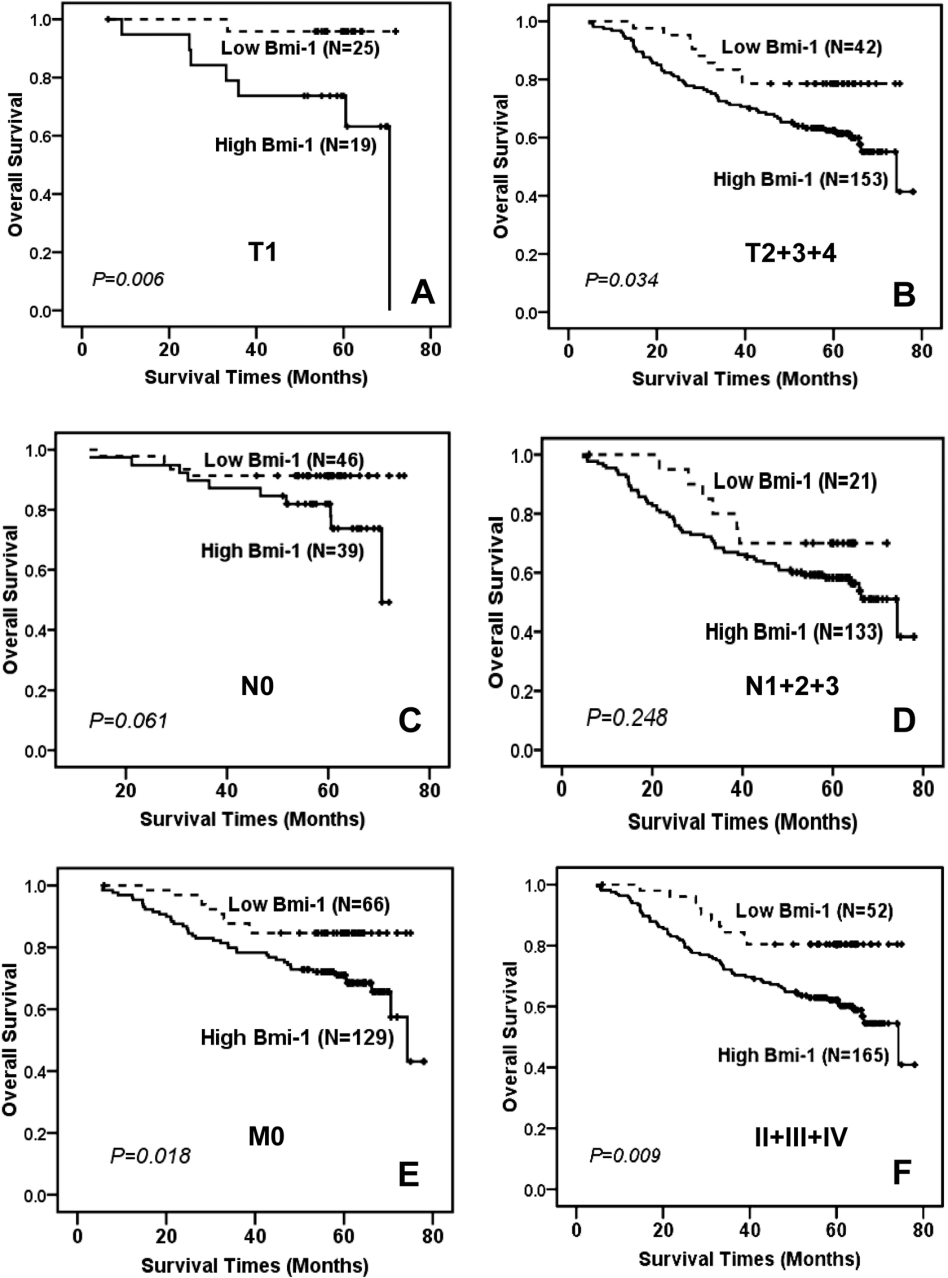
**Kaplan-Meier curves of overall survival stratified by Bmi-1 status according TNM classification**. **(A, B) **In each panel of patients with different tumor size, the overall survival was significantly shorter in the high Bmi-1 expression group than in the other group. **(C, D) **No obvious difference was seen according to the Bmi-1 expression in the N0 (**C**, *P = 0.061*) and N1+2+3 (**D**, *P = 0.248*) patients subgroups. **(E, F) **In the M0 panel and clinical stage II+III+IV panel, the overall survival was significantly shorter in patients with high Bmi-1 expression compared to those with low expression (**E**, *P = 0.018; **F**, P = **0.009).

As shown in Additional file 1, table S1, Bmi-1 expression displayed a significant correlation with patient survival status. The overall survival rate, assessed by the Kaplan-Meier method, was 85.1% (57 of 67) in the low expression group (mean follow-up period = 55.34 months), whereas it was only 59.9% (103 of 172) in the high expression group (mean follow-up period = 49.45 months) (Figure 2A). Because there were strong associations between the Bmi-1 status and clinicopathologic parameters, the overall survival might be further distinguished based on Bmi-1 expression and adjusting the status based on the clinicopathologic parameters. Consistent with previous data [47], 20.4% of the cases (30 of 147) displayed a prominent triple negative phenotype (TNP, ER^-^, PR^-^, and HER-2^-^). The outcome was not significantly different between the patients with high and low Bmi-1 expression (Figure 2B, *P = 0.483*). The overall survival rate in the TNP subgroup was 70% in the low expression group (7 of 10) compared to 75% (15 of 20) in the high expression group. Next, a subset analysis was carried out, in which we divided the patients into the ER negative and positive groups based on levels of Bmi-1 expression. Interestingly, according to the subset analysis, the impact on the outcome associated with high Bmi-1 expression continued to be more unfavorable in both the ER-negative and -positive groups (Figure 2 C, P = **0.003; D, P = *0.041). In ER-negative patients, the survival rate was 84.2% (32 out of 38) in patients with low Bmi-1 expression and 53.8% (43 out of 80) in those with high expression. Similarly, the survival rate was clearly different in the ER-positive subgroup. The survival rate was 86.2% (25 out of 29) in patients with low Bmi-1 expression and 65.2% (60 out of 92) in those with high expression. Similar results were obtained for the PR-negative and -positive groups (Figure 2 E, P = **0.010; F, P = *0.028). The survival rate in PR-negative patients was 79.3% (23 out of 29) in those with low Bmi-1 expression, in contrast to 47.4% (36 out of 76) in the high expression group. Likewise, the survival rate was 89.5% (34 out of 38) in those with low Bmi-1 expression compared to 69.8% (67 out of 96) in the high expression subset within the PR-positive subgroup. However, the overall survival was not obviously different based on Bmi-1 expression in the HER-2-negative subgroup (Figure 2G, *P = 0.701*), although the outcome was much better in patients with low Bmi-1 expression in the HER-2-positive subgroup (Figure 2H, P = *0.018). At the time of Bmi-1 analysis, 17.6% (3 out of 17) of HER-2-negative patients died with low Bmi-1 expression compared to 26.7% (8 out of 30) of HER-2-negative patients with high Bmi-1 expression. However, the survival rate was 88.9% (24 of 27) in the low Bmi-1 expression subset and 63.0% (46 of 73) in the high expression subset within the HER-2-positive subgroup. Because only a small number of cases showed low Bmi-1 expression and T3/4 classification (N = 9), N2/3 classification (N = 2), M1 classification (N = 2) and clinical stage III/IV (N = 6) (Table 3), the overall survivals were not analyzed stratified by these parameters. In addition, only 7 samples exhibited high Bmi-1 expression and stage I (Table 3), so the role of Bmi-1 in overall survival was not examined in the stage I subgroup. As shown in Figure 3, significantly different outcomes based on Bmi-1 expression were compared in patient subgroups with T1 (Figure 3A, P = **0.006) and T2+3+4 (Figure 3B, P = *0.034). When the tumor was less than 2 cm (T1 classification), the survival rate was 96.0% (24 of 25) in the low expression subset in contrast to 63.2% (12 of 19) in the high expression subset. Similarly, the survival rate was 78.6% (33 of 42) in the low expression subset compared to 59.5% (91 of 153) in the high expression subset when the tumors were larger than 2 cm (T2+3+4 classification). However, no obvious difference was observed when Bmi-1 expression was compared in the N0 and N1+2+3 subgroups (Figure 3 C, *P = 0.061*, D, *P = 0.248*). When the patients with an N0 classification were analyzed, the survival rate was 91.3% (42 of 46) in the low Bmi-1 expression group and 74.4% (29 of 39) in the high expression group. However, when the patients with N1+2+3 classifications were analyzed, the survival rate was 71.4% (15 of 21) in the low expression group and 55.6% (74 of 133) in the high expression group. In our study, 26.7% (52 of 195) of patients had died in the M0 group at the time of analysis, and the patients with low Bmi-1 expression showed longer survival times (Figure 3E, P = *0.018). The survival rate was 84.8% (56 of 66) in the low Bmi-1 expression group in contrast to 67.4% (87 of 129) in the high Bmi-1 expression group. A similar result was found in patients with stage II/III/IV, according to the Bmi-1 expression (Figure 3F, P = **0.009). The survival rate was 80.8% (42 of 52) in the low Bmi-1 expression subset compared to 58.8% (97 of 165) in the high Bmi-1 expression subset. Taken together, these results indicate that Bmi-1 could be helpful to evaluate the prognosis in patients with breast cancer.

### Analyses of relative risks (RRs) indicative of Bmi-1's role in the prognosis of breast cancer

In our analyses, we defined a relative risk of 1.000 as the baseline in patients with the following characteristics: age (≤45 years), T1, N0, M0, clinical stage I, low Bmi-1 expression and the absence of ER, PR and HER-2. To determine if Bmi-1 could serve as a risk factor with clinical usefulness, Cox regression proportional hazard analyses were used to examine the relative risk. As seen in Table 4, univariate Cox regression analyses revealed that a high level of Bmi-1 was associated with a significantly increased risk of death in breast cancer patients (***P = 0.001). The relative risk increased by almost 4-fold in patients with high Bmi-1 expression compared to those with low Bmi-1 expression (Table 4). As expected, large tumor size (T3, *P = 0.013; T4, ***P < 0.001), lymph node involvement (N1, P = **0.002; N2, ***P < 0.001), distant metastasis (M1, ***P < 0.001) and advanced clinical stage (III,*P < 0.025; IV, **P = 0.003) were also significant unfavorable prognostic factors. However, the presence of PR was a favorable prognostic factor (**P = 0.003), although the presence of ER and HER-2 did not predict the favorable or unfavorable survival (Table 4).

**Table 4:** Univariate Cox-regression analysis of different prognostic parameters in patients

	RR	95% CI	*P-value*
Age			
≤45 year	1.000		
>45 year	1.057	0.846-1.321	*0.624*
T Classification			
T1	1.000		
T2	1.534	0.713-3.301	*0.274*
T3	2.841	1.251-6.454	*0.013*
T4	7.822	3.294-18.861	*<0.001*
N Classification			
N0	1.000		
N1	2.597	1.431-4.713	*0.002*
N2	7.621	3.667-15.836	*<0.001*
N3	2.846	0.646-12.551	*0.167*
M Classification			
M0	1.000		
M1	3.039	1.903-4.853	*<0.001*
Clinical Stage			
I	1.000		
II	5.429	0.728-40.465	*0.099*
III	9.750	1.330-71.481	*0.025*
IV	20.065	2.724-147.736	*0.003*
ER Presence			
Negative	1.000		
Positive	0.758	0.486-1.183	*0.223*
PR Presence			
Negative	1.000		
Positive	0.511	0.327-0.800	*0.003*
HER-2 Presence			
Negative	1.000		
Positive	1.347	0.675-2.689	*0.398*
Bmi-1 Expression			
Low	1.000		
High	3.979	1.534-5.875	*0.001*

RR: Relative Risk; CI: Confidence Interval

The clinical stage, a comprehensive index reflecting T, N, and M classifications, is the most commonly used prognostic factor in the clinic. After adjustment for confounding factors, Bmi-1 was found to predict poor survival by multivariate Cox regression analyses when clinical stage, PR presence and Bmi-1 expression were included (*P = 0.042). Moreover, advanced clinical stage still predicted unfavorable prognosis (IV,*P = 0.014). PR was also identified as a potential prognostic factor by multivariate Cox regression analysis (**P = 0.007) (Table 5). Thus, our findings indicate that Bmi-1 protein expression has a significant correlation with the prognosis of breast cancer.

**Table 5:** Multivariate Cox regression analysis of potential prognostic factors for breast cancer patients

	RR	95% CI	*P-*value
Clinical Stage			
I	1		
II	4.378	0.584-32.793	*0.151*
III	6.322	0.832-48.209	*0.075*
IV	12.948	1.688-99.333	*0.014*
PR Presence			
Negative	1		
Positive	0.539	0.344-0.845	*0.007*
Bmi-1 Expression			
Low	1		
High	1.708	1.213-3.087	*0.042*

RR: Relative Risk; CI: Confidence Interval

### Exogenous expression of Bmi-1 enhances cell motility and invasion of immortalized HMECs

**Figure 4:**
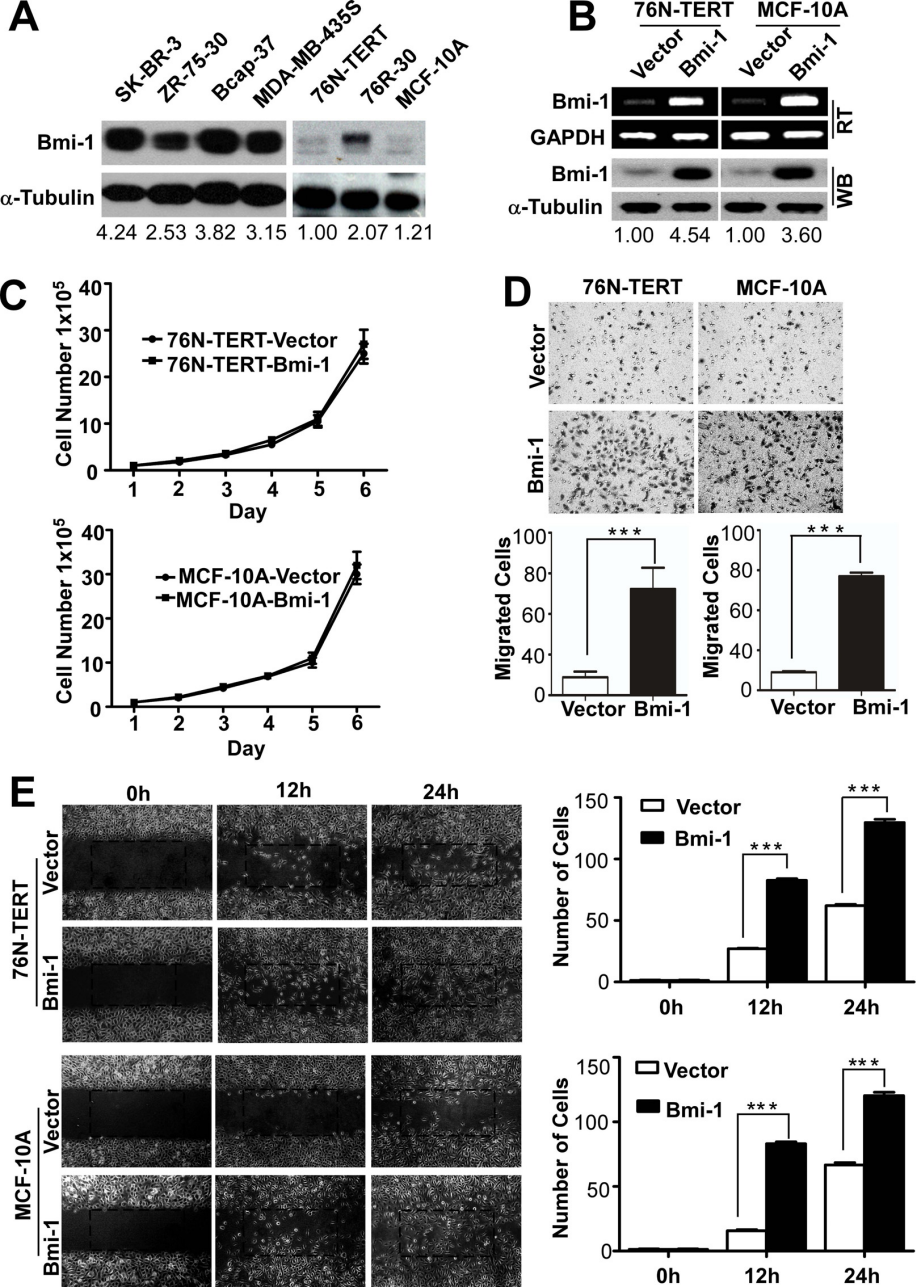
**The exogenous expression of Bmi-1 promotes the motility and invasiveness of immortalized HMECs**. **(A) **The endogenous expression of Bmi-1 was detected in breast cancer cells and immortalized HMECs by immunoblotting. **(B) **The overexpression of Bmi-1 in transfected immortalized HMECs was confirmed by RT-PCR and immunoblotting. GAPDH and anti-α-Tubulin were used as loading controls, respectively. **(C) **Bmi-1 did not affect the proliferation of immortalized HMECs. **(D) **The invasive properties induced by EGF and Insuline were analyzed by an invasion assay using Matrigel-coated Boyden chambers and scored under a light microscope (200X). **(E) **A wound was produced and monitored at 0, 12, and 24 hours as the cells moved and filled the damaged area in serum-free medium (200X). The data were plotted as the average number of cells per field of view (***P < 0.001).

Cell motility and invasion are indispensable for cancer metastasis. Because Bmi-1 expression was correlated with larger tumor size, lymph node involvement, distant metastasis and advanced clinical stage in breast cancer tissues, we hypothesized that Bmi-1 may regulate the progression of breast cancer. Because we were interested in the expression status of Bmi-1 in normal and breast cancer cells, western blotting was performed to measure Bmi-1 protein levels. Bmi-1 expression was low in p16-negative immortalized 76N-TERT and MCF-10A cells [13,47] and moderate in 76R-30 cells, whereas it was abundant in all breast cancer cell lines analyzed, including SK-BR-3, ZR-75-30, BCAP-37 and MDA-MB-435S (Figure 4A). To address the above-mentioned hypothesis, a Bmi-1 expression plasmid was stably transfected into immortalized HMECs (76N-TERT and MCF-10A) to examine the role of Bmi-1 in the progression of breast cancer (Figure 4B). Bmi-1 did not affect the proliferation of immortalized HMECs (Figure 4C). Boyden chamber and wound healing assays were performed to determine the potential for Bmi-1 to induce cell motility and invasion. The results showed that the overexpression of Bmi-1 increased cell invasion compared to the control (Figure 4D). Meanwhile, the overexpression of Bmi-1 could advance the wound healing process, by promoting the quicker closure of a ''wound'' scratched into a confluent epithelial monolayer (Figure 4E). Pooled populations of cells expressing Bmi-1 or vector were analyzed for a transformed phenotype using soft agar and Matrigel assays. The 3-D Matrigel assay indicated that the expression of Bmi-1 failed to transform the morphology of immortalized HMECs. No irregular branched structures indicative transformed phenotypes were observed, other than normal spherical acini (data not shown). To further confirm the in vitro transformation potential, the immortalized HMEC-derived cells were seeded in soft agar. Cells expressing either Bmi-1 or vector did not exhibit anchorage-independent growth (data not shown). These observations indicate that Bmi-1 does promote cell motility and invasion, but Bmi-1 alone is insufficient to transform immortalized HMECs.

### Suppression of Bmi-1 represses cellular motility, invasion and transformation

**Figure 5:**
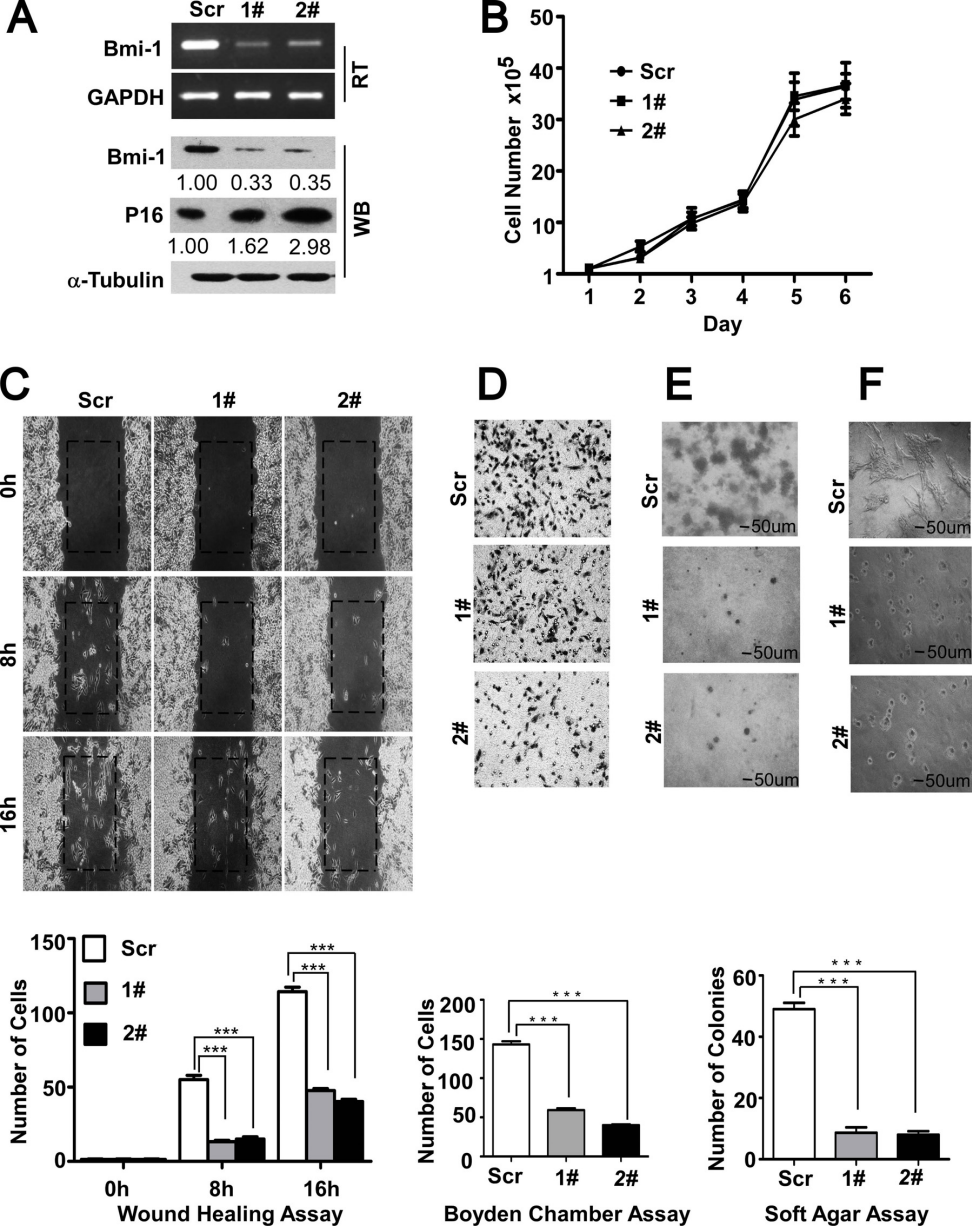
**Suppression of endogenous Bmi-1 inhibits cellular motility, invasion and transformation**. **(A) **Bmi-1 expression was confirmed by RT-PCR and immunoblotting. It was dramatically decreased by RNA interference in MDA-MB-435S cells. **(B) **The Bmi-1 knockdown did not alter the proliferation of MDA-MB-435S. **(C) **The mobility was measured by testing the rate of wound closure at 0, 8, 16 hours (200X). **(D) **The invasive properties induced by FBS were analyzed by using the Matrigel-coated Boyden chamber assay (400X, ***P < 0.001). **(E) **Anchorage-independent growth was measured in soft agar (200X, ***P < 0.001). **(F) **The acini formation of cells was tested in Matrigel culture (200X).

To further identify the role of Bmi-1 in the progression of cancer, a short hairpin RNA for Bmi-1 was generated to reduce Bmi-1 expression stably and efficiently in the MDA-MB-435S cell line (Figure 5A), a highly metastatic breast cancer cell line [48] with high Bmi-1 expression (Figure 5A). As expected, p16INK4, a Bmi-1 target gene [21], was up-regulated in the Bmi-1 knockdown cells. However, the proliferation rate did not show an obvious alteration in response to Bmi-1 repression (Figure 5B). The Boyden chamber invasion assay and the scratch wound healing assay revealed that the motility and invasiveness of MDA-MB-435S cells were dramatically hampered by the ablation of Bmi-1 (Figure 5C, D). In addition, the growth of colonies in soft agar, as an indication of in vitro cellular transformation, were less in frequent and smaller in size, which indicated that the depletion of Bmi-1 caused the marked inhibition of anchorage-independent growth ability (Figure 5E). Furthermore, Bmi-1 repression caused the disappearance of the irregular, branched structures in Matrigel cultures, which characterize the invasive phonotype (Figure 5F). Our results suggest that the repression of Bmi-1 could decrease cell motility, invasion and transformation.

### Repression of Bmi-1 slows tumor progression and reduces spontaneous lung metastasis in nude mice

**Figure 6:**
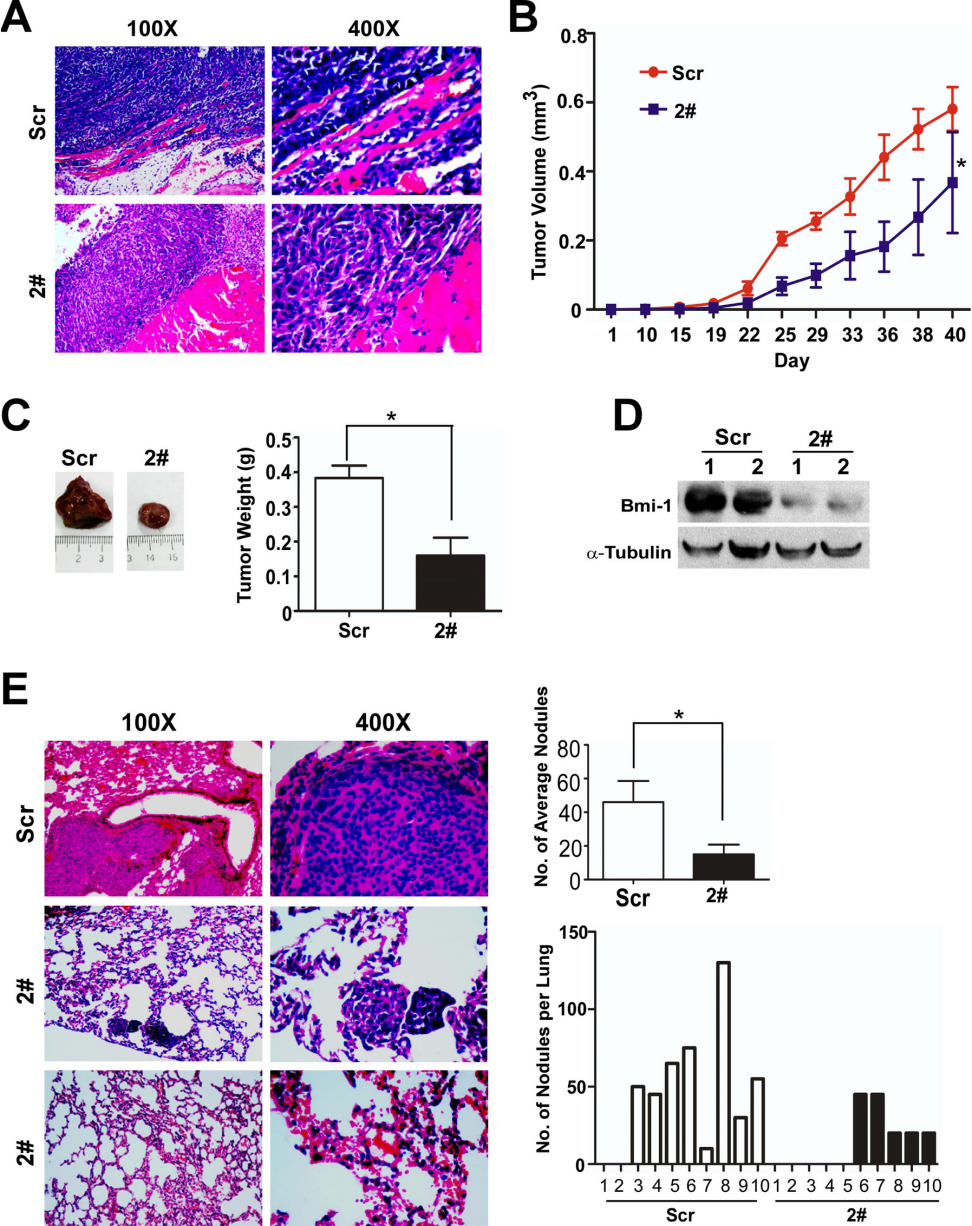
**Suppression of endogenous Bmi-1 slows down tumorigenesis and decreases lung metastasis in nude mice**. **(A) **H&E staining confirmed the invasiveness of the primary xenografts by analyzing tumor cell encroachment into adjacent muscle (100X and 400X). **(B) **The initiation and growth of primary xenografts were determined by measuring the average tumor volume (*P = 0.038), **(C) **the tumor size and average weight of primary xenografts (*P = 0.041). **(D) **Bmi-1 expression in the primary xenografts was detected by immunoblotting (1 and 2 were abbreviated from sample 1 and sample 2). **(E) **The number of spontaneous lung metastatic lesions in mice (N = 10 per group) was analyzed by counting ten serial sections from each sample (*P = 0.036).

To further evaluate the effects of Bmi-1 on the development of breast cancer, MDA-MB-435S/shBmi-12# and MDA-MB-435S/shScr cells were injected into the fat pad of nude mice. Macroscopic xenografts were observed in the fat pad of nude mice after two weeks. The tumors arising from injection of MDA-MB-435S/shBmi-12# cells were histologically similar to those from controls, as assessed by hematoxylin and eosin staining and reviewed by a veterinary pathologist (Figure 6A, D). The xenografts from MDA-MB-435S/shScr cells invaded the adjacent muscles deeply, whereas, the MDA-MB-435S/shBmi-12# cells showed reduced invasiveness (Figure 6A). The repression of Bmi-1 not only reduced the volume and weight of the xenografts but also delayed tumor occurrence (Figure 6B, C). Western blotting confirmed the persistent knockdown of Bmi-1 in the xenograft tissues (Figure 6D). Necropsy revealed large fulminant gross metastatic lesions in the lungs, involving large portions of all lung lobes in eight out of ten mice injected with the MDA-MB-435S/shScr cells. In contrast, only small and limited metastatic lesions were observed in the lungs of five out of ten mice injected with the MDA-MB-435S/shBmi-12# cells (Figure 6E). However, injection of MCF-10A/Bmi-1 cells neither formed xenografts in the fat pad nor caused metastatic lesions in nude mice, even if SCID mice were used (data not shown). These results indicated that overexpression of Bmi-1 was not sufficient for the fully malignant transformation of immortalized HMECs, whereas knockdown of Bmi-1 strongly slowed tumor progression and repressed spontaneous lung metastasis in nude mice.

### The expression of epithelial and mesenchymal markers was altered by Bmi-1

**Figure 7:**
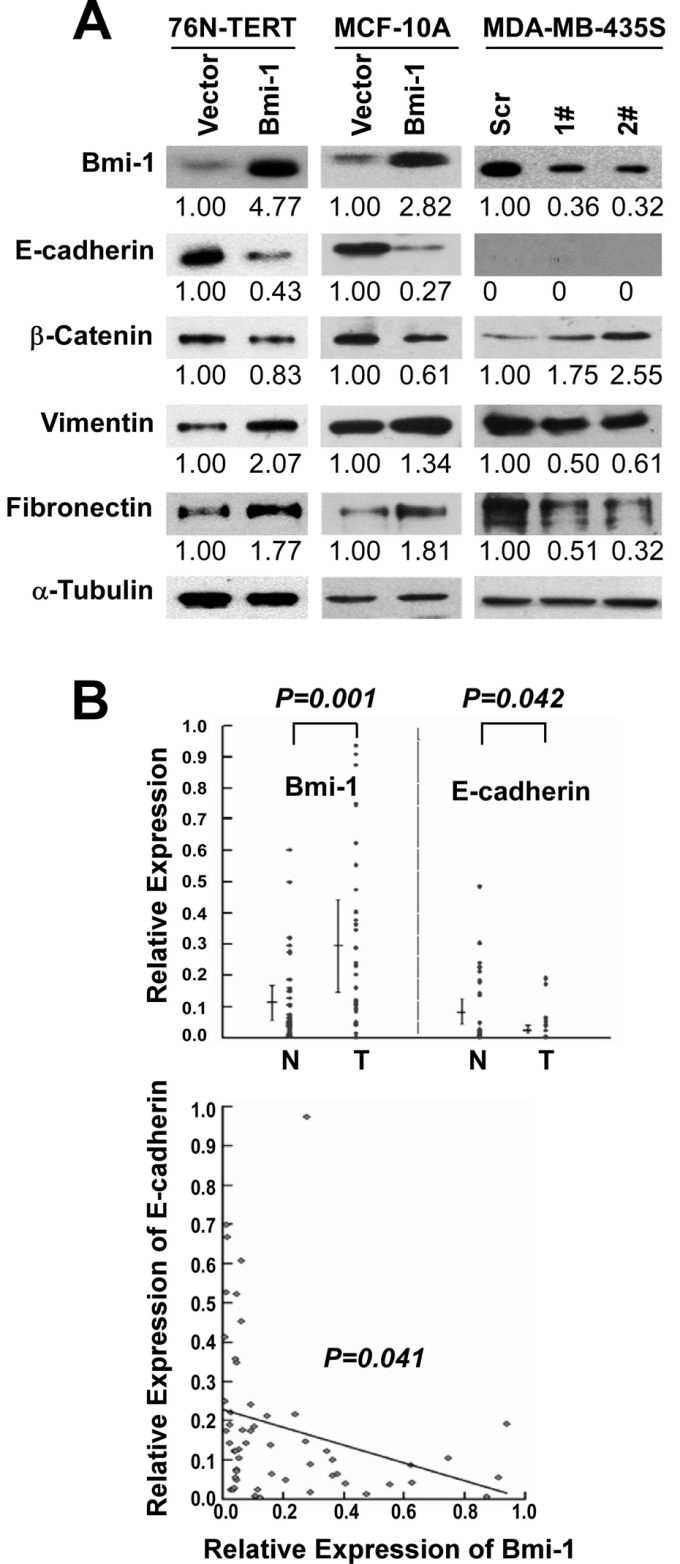
**Bmi-1 regulates EMT markers**. **(A) **The expression of the indicated proteins was analyzed by immunoblotting. Anti-α-Tubulin was the loading control. **(B) **Top: The mRNA of Bmi-1 and E-cadherin were compared between the breast cancer tissues and the adjacent non-cancer tissues (**P = 0.001, *P = 0.042). Bottom: The converse relationship between Bmi-1 and E-cadherin was plotted (Spearman's rho = -0.418,*P = 0.041).

**Figure 8:**
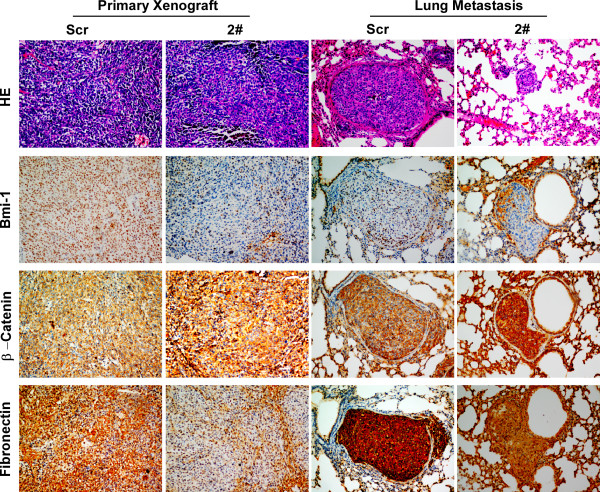
**Suppression of endogenous Bmi-1 reversed EMT markers in nude mice**. H&E staining indicated the similarity of histopathology of primary xenografts and lung metastases in two groups of mice. The indicated proteins were analyzed by immunohistochemistry. Bmi-1 and Fibronectin detected in both primary xenografts and lung metastasis were down-regulated, whereas, β-Catenin was up-regulated in the MDA-MB-435S/shBmi-12# group compared with the MDA-MB-435S/shScr group.

The expression of EMT markers was analyzed to address the mechanism of Bmi-1-facilitated breast cancer metastasis. Although no EMT-associated morphological changes were observed in Bmi-1 overexpressing and knockdown cells, overexpression of Bmi-1 repressed epithelial markers, such as E-cadherin and β-Catenin, and up-regulated mesenchymal markers such as Vimentin and Fibronectin. Conversely, the knockdown of Bmi-1 inhibited the expression of Vimentin and Fibronectin but partially rescued the expression of β-Catenin (Figure 7A). E-cadherin was not detected in MDA-MB-435S cells in the present study, owing to its unique properties [49]. To further validate the role of Bmi-1 in EMT, mRNA levels of Bmi-1 and E-cadherin were measured in 34 breast cancer tissues and in paired non-cancerous tissues from the same patients by real-time PCR. As shown in Figure 7B, Bmi-1 was strongly up-regulated in breast cancer tissues compared with paired non-cancerous tissues, whereas E-cadherin was markedly down-regulated. Additionally, an inverse correlation was found between Bmi-1 and E-cadherin at the transcriptional level. To further decipher the role of Bmi-1 in the invasion and metastasis of breast cancer, EMT markers were analyzed in primary xenografts and spontaneous metastatic lung lesions by immunohistochemistry. As shown in Figure 8, Bmi-1 repression enhanced the expression of β-Catenin and concomitantly reduced the expression of Fibronectin in primary xenografts and metastatic lung lesions. As demonstrated above, Bmi-1 is negatively correlated with the expression of E-cadherin, which is important for EMT in breast cancer.

### Bmi-1 activates the Akt/GSK-3β/Snail pathway

**Figure 9:**
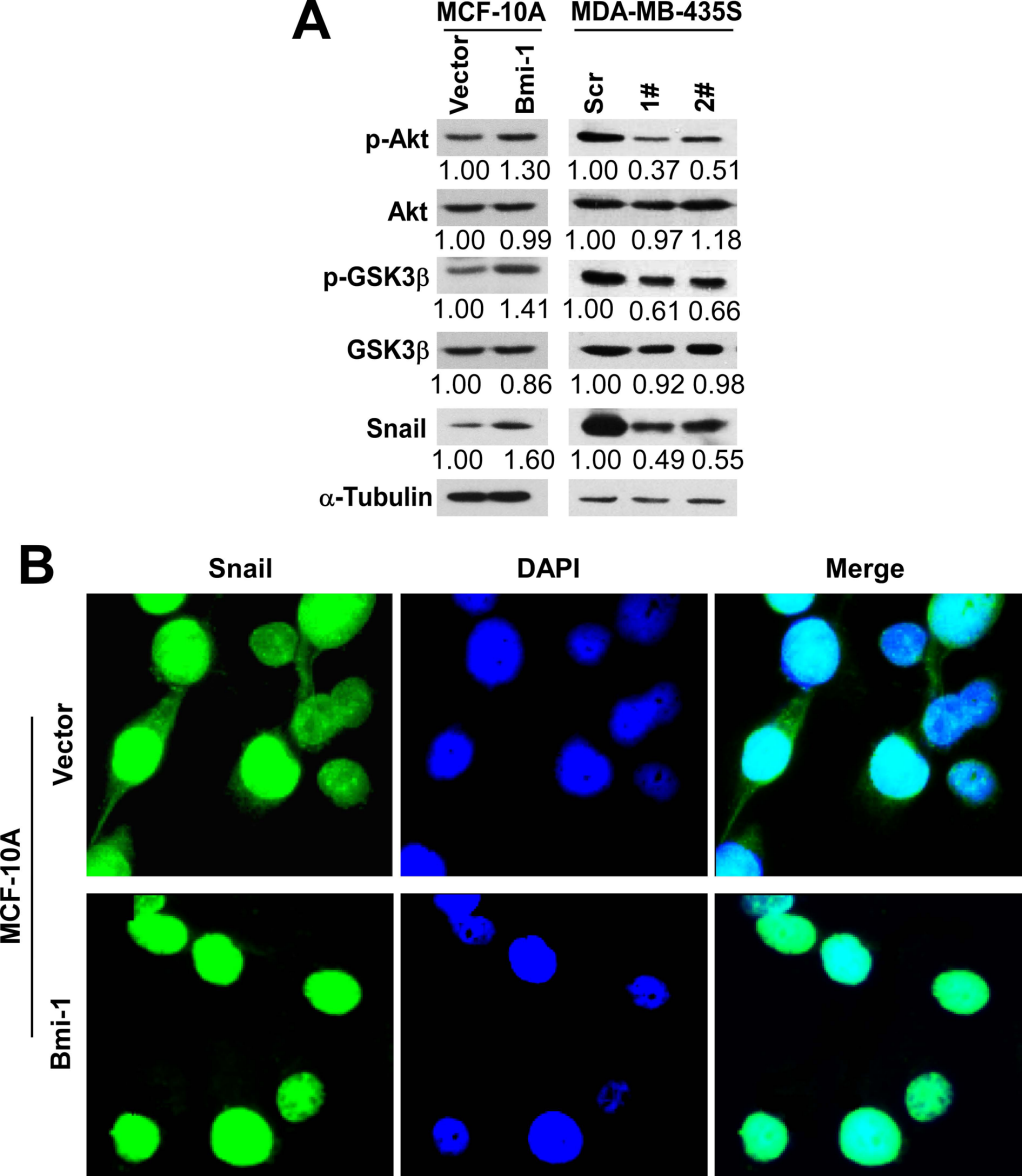
**Bmi-1 modulates Akt/GSK3β/Snail pathway**. **(A) **Cell extracts were analyzed by immunoblotting with antibodies against the indicated proteins. Anti-α-Tubulin was used as the loading control. **(B) **Immunofluorescence identified the effect of Bmi-1 on the subcellular localization of Snail proteins (Green) in MCF-10A cells.

Consistent with our previous reports that Bmi-1 could regulate Akt activity in breast cancer cells [50,51] and the Akt/GSK-3β/Snail pathway in NPC cells [27], the overexpression of Bmi-1 facilitated the expression of phosphorylated Akt. Moreover, the knockdown of Bmi-1 inhibited the expression of phosphorylated Akt, but total Akt remained unaffected (Figure 9A). As anticipated, the expression of Snail and phosphorylated GSK-3β was up-regulated by Bmi-1 overexpression and down-regulated by Bmi-1 knockdown, but the levels of total GSK-3β remained unaffected (Figure 9A). Nevertheless, the transcriptional level of Snail was not affected by Bmi-1 overexpression (data not shown), suggesting that the modulation of Snail might be due to a post-transcriptional modification. Bmi-1 could extend the half-life of Snail in NPEC cells by directing the subcellular localization, as demonstrated by our previous data [27]. Therefore, we analyzed the localization of Snail in MCF-10A cells. As shown in Figure 9B, Snail could be detected in the nucleus and cytoplasm of the controls, but it was primarily localized in the nucleus of the Bmi-1-transfected cells. Collectively, it appears that Bmi-1 induces the activation of Akt and the inactivation of GSK-3β by phosphorylation, facilitates the stabilization and nuclear translocation of Snail, and finally results in the deregulation of EMT markers, thus promoting the migration and invasion of breast cancer cells.

## Discussion

Breast cancer, a common malignant disease in women, is prone to invade into adjacent regions and to metastasize to lymph nodes and distant organs. To develop novel treatments and cures, it is imperative to address the factors underlying tumorigenesis, invasion and metastasis. In this study, we identified and functionally characterized Bmi-1 as an important player in breast cancer progression. The current study first illustrated the expression of Bmi-1 in primary breast cancer tissues, followed by demonstrating the association between the Bmi-1 expression and clinicopathologic parameters and finally addressed the role of Bmi-1 in breast cancer prognosis in a large series of 252 samples. In this study, differential expression of Bmi-1 was detected between primary cancer tissues and the matched adjacent non-cancerous tissues. Bmi-1 expression was significantly up-regulated in breast cancer tissues compared with the adjacent non-cancerous tissues, which was echoed by our previous reports [15,50]. Only 35.9% of matched adjacent non-cancerous tissues displayed the high Bmi-1 expression, whereas as many as 72.2% of the cancer tissues were defined by high Bmi-1 expression. The incidence of high Bmi-1 expression shown in this study was much higher than previously demonstrated (53.2%) [19]. The difference may reflect differences in Bmi-1 status in the samples used in different studies, which obtained tissue samples from patients with different disease stages, or samples from different populations. As shown in Additional File 2, table S2, 46.4% of the Chinese samples recruited in our study were from the early stage (I,II), while 75.8% of Korean samples used in Choi's study were from the early stage (I,II)[19]. In addition, no samples of stage □ were recruited in Choi's study [19]. Furthermore, the difference may come from variations in antigen retrieval, antibody dilution, development time and the positive criteria adjusted, especially the score of the positive number (Additional File 3, 4, 5, Table S3,S4,S5). For example, we used EDTA buffer to retrieve antigen in our research, as opposed to citrate buffer used in Choi's study [19]. In addition, the development time in our study was 10 min compared with 5 min in Choi's study [19]. The criteria used in the immunohistochemistry varied in different studies. Choi *et al.* tested Bmi-1 expression by tissue microarray, which might not be a good representative of the whole paraffin-embedded tissue. Furthermore, cells were considered positive for Bmi-1 only when nuclear staining was observed [19]. However, both nuclear and cytoplasm staining were observed in our samples. However, to further confirm Bmi-1 expression in breast cancers, multi-center studies are required.

We also compared the Bmi-1 mRNA expression in 34 paired tissues, including breast cancer and matched adjacent non-cancerous tissues. There was a significant differential between the breast cancer tissues and the adjacent non-cancer tissues, which corresponded to the protein levels in the tissues. In our analysis, high Bmi-1 expression showed an obvious correlation with larger tumor size, lymph node involvement, organ metastasis and advanced clinical stage. Over 90% of the late-stage (stages III/IV) samples showed high expression, whereas less than 50% of the early-stage (stagesI/II) samples showed high expression. These results revealed that higher Bmi-1 expression was related to more aggressive behavior, which was further supported by its expression in distant metastases. Of patients without distant metastasis, 67.1% showed high Bmi-1 expression, which is in contrast to 95.6% of patients with distant metastasis. These studies indicated that a high level of Bmi-1 protein might contribute to the invasion and progression of breast cancer. Although only 35.9% of the adjacent non-cancerous tissues displayed high Bmi-1 expression, 69.2% stained positive for Bmi-1. Because hyperplasia is known to occur often in adjacent non-cancerous tissues, we speculated that the Bmi-1 staining had originated from hyperplasia.

In this study, the Bmi-1 protein seemed to localize in the nucleus of the breast cancer cells and in the cytoplasm of the non-cancer cells. It has been reported that phosphorylation can explain differential subcellular localization of some of the polycomb family genes, such as Nervous System Polycomb 1(NSPc1) and M33 [52,53]. Previously, it has been reported that there is a rich proline/serine region at the carboxyl terminus of the Bmi-1 protein, where phosphorylation often occurs [54]. However, whether the phosphorylation of Bmi-1 is a direct cause or merely associated with the nuclear-cytoplasm shuttling events remains to be determined. It is also important to note that Bmi-1 predicted poor prognosis in breast cancer, in accordance with other reports [21,24,55-58]. In addition, there were significantly different outcomes between the patients expressing high and low levels of Bmi-1 by subset analysis, which suggested that Bmi-1 may be used to predict the clinical outcome. In addition, it provides a potential therapeutic target for the future treatment of breast cancer.

In our study, Bmi-1 was not significantly correlated with ER and PR expression, which is consistent with a previous report indicating that Bmi-1 mRNA expression had no significant correlation with ER or PR expression [20], but it is inconsistent with other previously-published data [15,18,59]. Nevertheless, a statistically significant association was observed between Bmi-1 expression and survival when ER or PR was included in our analysis. ER^+ ^cancer cells depend on estrogen for their growth, so they can be treated with drugs that block the effect of estrogen. Patients with ER present were offered adjuvant hormone therapy in our study. High Bmi-1 expression was associated with unfavorable survival, irrelevant to ER or PR presence, indicating that hormonal therapy did not affect the prognostic role of Bmi-1. Patients with metastatic breast cancer may take tamoxifen for varying lengths of time, depending on the cancer's response to this treatment and other factors [60]. When used as adjuvant therapy for early-stage breast cancer, tamoxifen is generally prescribed for five years. However, the ideal length of treatment with tamoxifen is not known. Thus, different lengths of treatments with tamoxifen depending on the individual responses to it may have resulted in a different outcome in our study. In addition, the combination of endocrine therapy with other therapy remains a research issue [61]. Generally, patients who are positive for HER-2 had a worse prognosis [62]. However, these 252 patients did not receive anti-HER-2 therapy because Herceptin (a humanized monoclonal antibody directed at the HER2 ectodomain) was not in use at that time in China. Although there was no significant correlation between Bmi-1 expression and HER-2 status, patients with high Bmi-1 expression showed poor survival stratified by HER-2, just as with ER and PR. Having demonstrated the significance of Bmi-1 in the overall survival prognosis of breast cancer patients, it will be our next focus to investigate the prognostic value of Bmi-1 in terms of disease-free survival and cancer-specific survival. Additionally, a possible correlation between Bmi-1 expression and outcome after hormonal therapy and chemotherapy warrants investigation and would require a large number of samples.

Metastatic relapse remains a major challenge in breast cancer management. Many factors are involved in tumor progression, including changes in cell adhesion, cell communication, increased migration or motility and invasiveness [63]. In this study, Bmi-1 was shown to contribute to each of these events in clinical samples and in cell lines. To address the role of Bmi-1 in tumor progression, Bmi-1 was overexpressed in two immortalized HMEC lines, 76N-TERT and MCF-10A. Conversely, RNA interference was used to decrease the expression of Bmi-1 in MDA-MB-435S, an estrogen-independent breast cancer cell line derived from a mammary ductal carcinoma [64]. MDA-MB-435S cells can form progressively growing tumors in the lungs and regional lymph node metastases following injection into the mammary fat pad of 3-4 week old athymic nude mice [65]. In this study, the overexpression of Bmi-1 alone could not fully transform 76N-TERT or MCF-10A cells. Furthermore, Bmi-1 expression did not alter the morphology of these cells in 3-D Matrigel culture. In addition, the spindle-shaped phenotype and non-contact inhibited, disorganized proliferation of MDA-MB-435S cells [66] was not altered by the inhibition of Bmi-1. This result was in accordance with a previous observation that Bmi-1 alone did not result in an EMT phenotype in MCF-10A cells, but that co-overexpression of Bmi-1 and Ras readily did [28]. Additionally, we examined the potential oncogenic role of Bmi-1 by the injection of Bmi-1-expressing MCF-10A cells into mice. Even injection of 1 × 10^7 ^MCF-10A/Bmi-1 cells did not result in tumor formation after two months in nude or SCID mice. Unlike in immortalized NPECs, Bmi-1 alone was not sufficient to induce the typical EMT morphological changes in immortalized HMECs. The induction of morphological alterations associated with EMT by Bmi-1 might depend on the cell type. To our knowledge, the immortalized NPECs were derived from squamous epithelium, whereas the immortalized HMECs originated from glandular epithelium. In addition, the morphologic changes of EMT might be directed by differential oncogene activation. Ras [67] and ILEI [68] can lead to EMT, tumor formation and metastasis. These results suggest that additional oncogenic events, such as H-Ras expression or loss of expression of tumor suppressor genes could be involved in the EMT of immortalized HMECs induced by Bmi-1. Thus, we suggest that Bmi-1-induced EMT is cell-type specific.

One thing worth mentioning is that although E-cadherin, a useful molecule to protect breast cancer from metastasis [69], was not detected in MDA-MB-435S cells, the MDA-MB-435S/shBmi-1 cells still manifested reduced motility. To our knowledge, several highly metastatic cancer cells, including MDA-MB-435S cells, lack E-cadherin expression [70]. Low E-cadherin expression can be caused by gene mutations or promoter methylation [71,72], as well as by regulation by inhibitors such as Twist [73]. After EMT, mesenchymal FosER cells completely lacked E-cadherin but formed neither tumors nor metastases [74], indicating that loss of E-cadherin expression might be necessary but not sufficient for tumor progression. Similarly, although E-cadherin expression was decreased by Bmi-1 overexpression, the HMECs did not form tumors in the current study. As we know, besides E-cadherin, many other genes are involved in breast cancer metastasis, such as β-Catenin [75] and N-cadherin [76]. Numerous studies have linked aberrant E-cadherin with the development of metastasis in cancer [77], whereas other studies have presented different results indicating that cells from distant metastases and nodal involvement consistently expressed E-cadherin, often at higher levels than in the primary tumor [78,79]. It appears that translational regulation and post-translational events are probable mechanisms for E-cadherin re-expression [80]. It is possible that loss of E-cadherin is a transient phenomenon that allows malignant cells to invade vascular channels and tissues. Disseminated mesenchymal cancer cells seem to undergo the reverse transition, mesenchymal-epithelial transition (MET), at the metastatic site to allow micrometastases to give rise to a secondary neoplasm. In this regard, cancer cells from the secondary site re-express markers of epithelial cells such as E-cadherin. However, whether re-expression of E-cadherin occurs in Bmi-1 overexpressing cancer cells in metastatic site, and if so, what is the underlying mechanism requires further investigation. In addition, our data suggest that Bmi-1 has a critical effect on breast cancer tumorigenesis and lung metastasis. We believe that this is an extremely important observation in terms of studying breast cancer lung metastasis because the lung is the most common location of breast cancer metastasis. We suggest that Bmi-1 contributes to the metastasis of breast cancer. Crosstalk between different pathways, recognized as a mechanism for expanding the cellular communication signaling network, is currently receiving increased attention. The activated PI3K/Akt pathway has been well documented in various human malignancies and sometimes correlates with an aggressive phenotype [81]. Our previous data also indicated that down-regulation of Bmi-1 by an RNA interference (RNAi) approach was accompanied by down-regulation of Akt/protein Kinase B (PKB) activity [50]. In our current study, we demonstrated that Bmi-1 induced invasion, which might be associated with activation of the Akt pathway in breast cancer cells. As metastasis can occur in early stages of tumor development, some genes may constantly regulate tumor development. They may not only facilitate primary tumor initiation but also promote tumor transformation and metastasis [82]. The expression pattern of Bmi-1, together with functional studies, indicate that Bmi-1 plays a prominent role in breast cancer progression and metastasis and opens the door for future studies addressing Bmi-1-targeted therapy in breast cancer.

## Conclusions

In summary, breast cancer shows a high prevalence of Bmi-1 expression, which is significantly correlated with aggressive features and unfavorable prognosis. Assessment of Bmi-1 expression might help to identify a high-risk subgroup of breast cancers. Furthermore, Bmi-1 plays a crucial role in invasion and metastasis by modulating the Akt/GSK-3β/Snail pathway and the expression of EMT markers in breast cancer.

## Materials and methods

### Tissue Samples

Paraffin-embedded breast cancer samples were obtained from 252 female Chinese patients (median age: 47 years, range: 26-78 years) diagnosed with breast cancer in 1999-2001 at Cancer Center, Sun Yat-sen University, Guangzhou, China. Of the 252 breast cancer samples, 39 matched adjacent non-cancerous tissues were obtained from the above-mentioned patients. All of the samples were treated by surgical excision. Among them, 239 cases had follow-up records and the median follow-up time was 59 months (range: 4-78 months). Clinical and pathologic factors were evaluated, including age, TNM classification, clinical stage, presence of steroid receptors and HER-2 expression. HER-2 expression was only analyzed in 159 cases, while the other samples were not analyzed. A total of 147 cases were analyzed for ER, PR and HER-2 expression. Thirteen cases were missing records of patient age, survival time and survival status, but included the TNM classification, clinical stage, presence of steroid receptors and HER-2 expression. To use these clinical materials for research purposes, prior patients' consent and approval from the Institute Research Ethics Committee were obtained. The observation period was from 1999 to 2006. The clinical stages of all the patients were classified according to the 2002 TNM staging of UICC (International Union against Cancer).

### Immunohistochemistry in Clinical Samples

The 4 μm paraffin-embedded sections of breast cancer were deparaffinized with xylene, rehydrated and treated with 3% hydrogen peroxide in methanol to quench the endogenous peroxidase activity. Subsequently, antigen retrieval was performed by heating in a microwave oven with EDTA (pH 8.0). One percent bovine serum albumin (BSA) was used to block non-specific binding, followed by incubation of the sections with a mouse monoclonal anti-Bmi-1 antibody (1:100, Upstate Biotechnology, Lake Placid, USA) overnight at 4°C. After washing with phosphate buffered saline, sections were incubated with biotinylated secondary antibody, followed by a further incubation with the streptavidin-horseradish-peroxidase complex. The sections were then immersed in 3, 3'-Diaminobenzidine (DAB) for 10 min, counterstained with 10% Mayer's hematoxylin, dehydrated, and mounted in crystal mount. The primary antibody was replaced by non-immune mouse IgG of the same isotype to serve as negative controls. To minimize variations in the immunopositive cells, all sections were stained in DAB for the same amount of time. Two pathologists, blinded to the clinical outcome, scored the results of the staining independently. Measurements of ER, PR and HER-2 were routinely performed as previously described [83].

### Cell lines, Vectors and Plasmids

Immortalized HMECs (76N-TERT and MCF-10A) and radiation-transformed cells (76R-30) were cultured in Keratinocyte-SFM medium (Invitrogen, Grand Island, NY) supplemented with bovine pituitary extract. MDA-MB-435S cells were maintained in DMEM/F12 (Gibco) supplemented with 10% fetal calf serum (HyClone). SK-BR-3, ZR-75-30 and BCAP-37 cells were grown in RPMI 1640 with 10% fetal calf serum. The pMSCV-Bmi-1 and Bmi-1 short hairpin RNA (shRNAi) constructs were generated as described previously [21,51]. Retrovirus expressing Bmi-1 was produced and transfected into 76N-TERT and MCF-10A cells, as described previously [13]. The plasmid with shBmi-1 was introduced into MDA-MB-435S cells, which showed strong ability to metastasize [48]. The sequences of shRNA were as follows: shBmi-1 1# GUUCACAAGACCAGACCAC and shBmi-1 2# GACCAGACCACUACUGAAU [51]. pMSCV and PRS plasmids were used as controls. All retrovirally infected cells were maintained under Puromycin selection and used as stable cells.

### RT-PCR, Real-time PCR and Western Blot Analysis

Total RNA from fresh tissues and cell lines was isolated using Trizol reagent (Invitrogen, Grand Island, NY), according to the manufacturer's instructions, and 1.0 μg of total RNA treated with DNAase was used for cDNA synthesis by random hexamers. Genes were amplified by PCR from cDNA. The forward primer for Bmi-1 was 5-'CTGGTTGCCCATTGACAGC'-3, the reverse primer was 5-'CAGAAAATGAATGCGAGCCA'-3. The forward primer for GAPDH was 5-'AGCCGTTCGGAGGATTATTCG'-3, the reverse primer was CTTCTCCTCAGCAGCCAGAG. The products were analyzed by agarose gel electrophoresis and confirmed by appropriate size.

Real-time PCR was carried out using an ABI PRISM 7500 Sequence Detection System (Applied Biosystems, Foster City, CA). Reactions were performed in triplicate repeats in two independent experiments. The geometric mean of the GAPDH (glyceraldehyde-3-phosphate dehydrogenase) housekeeping gene was used as an internal control to normalize the variability in expression levels. The forward primer for Bmi-1 was 5-'CTGGTTGCCCATTGACAGC'-3, the reverse primer was 5-'CAGAAAATGAATGCGAGCCA'-3 and the probe was FAM-CAGCTCGCTTCAAGATGGCCGC-TAMRA. The forward primer for E-cadherin was 5-'GAACAGCACGTACACAGCCCT'-3, the reverse primer was 5-'GCAGAAGTGTCCCTGTTCCAG'-3 and the probe was FAM-ATCATAGCTACAGACAATGGTTCTCCAGTTGCT-TAMRA. The forward primer for GAPDH was 5-'GACTCATGACCACAGTCCATGC'-3, the reverse primer was 5-'AGAGGCAGGGATGATGTTCTG'-3 and the probe was FAM-CATCACTGCCACCCAGAAGACTGTG-TAMRA.

Immunoblotting was carried out as described [84]. The blots were probed with mouse anti-Bmi-1, anti-E-cadherin, anti-β-catenin, anti-fibronectin and anti-vimentin antibodies (BD, Transduction Laboratories, Lexington, UK) as well as with rabbit anti-p-GSK, anti-t-GSK (Cell Signaling Technology, Inc. USA), anti-snail (Abcam, Cambridge Science Park, Cambridge, UK), anti-p-Akt (Santa Cruz Biotechnology, CA, USA) and goat anti-t-Akt (Santa Cruz Biotechnology, CA, USA) antibodies. The membranes were stripped and re-probed with mouse anti-α-tubulin (Sigma Aldrich, Inc. St Louis, Missouri, USA) to confirm equal loading of the samples.

### Wound Healing Assay

Cells were seeded in six-well plates and cultured under permissive conditions until 90% confluence. After starving the cells for 24 h in medium without EGF or FBS, the confluent cell monolayer was lightly and quickly scratched with a pipette tip to produce a straight line. The debris was removed and the edge of the scratch was smoothed with PBS washing. The wound healing assays were done in growth factor-free medium, further excluding any effect due to a potential proliferation difference. The open gap was then inspected and photographed microscopically at indicated times, and is shown in the Figures at a 200X magnification. The migration activity was calculated as the number of cells entering into the rectangle. Experiments were repeated a minimum of three times.

### Proliferation Assay

1 × 10^5 ^cells were plated on a P60 plate. Every 24 h, cells were trypsinized and counted under a light microscope at least three times until the sixth day. Experiments were repeated a minimum of three times.

### Boyden Chamber Assay

This assay measures the ability of cells to invade a Matrigel matrix overlying a membrane containing 8-μm pores. Cells were seeded in medium deprived of EGF or FBS in the top chamber (BD), whereas medium containing EGF or FBS was added to the bottom chamber. After an appropriate cultivation time, the chambers were fixed with 1% paraformaldehyde and stained with hematoxylin. The number of cells in ten random fields of view was enumerated at 200X or 400X magnification for each filter. Three independent experiments were performed and the data are presented as the mean ± SD.

### Three Dimensional Matrigel Culture

Matrigel (1.2 mg/ml, BD) was coated on the bottom of a 24-well plate. After Matrigel polymerization, cells were seeded into the well with growth medium containing 2% Matrigel. The cells were cultivated at 37°C incubation and alterations to the morphologic phenotype were monitored at 200X magnification every other day. Experiments were repeated a minimum of three times.

### Anchorage-Independent Growth in Soft Agar

The soft agar assay was used to determine the propensity for anchorage-independent growth. Cells were plated in a 60-mm dish using 2 ml of growth medium, including 0.33% agar on the top of a bottom layer containing 0.66% agar. The cells were fed every two days with 1 ml medium. Colonies were photographed and counted in ten random fields of view at 200X magnification using light microscopy. Each experiment was done in triplicate.

### Confocal Immunofluorescence Microscopy

Cells were seeded onto glass slides for 24 h, washed with PBS, fixed in 4% paraformaldehyde and permeabilized with 0.5% Triton X-100 for five minutes. After blocking with BSA, cells were stained with anti-snail primary antibody followed by FITC-conjugated anti-rabbit IgG. To visualize the nucleus, 4' 6-Diamidino-2-phenylindole (DAPI) staining was also performed, as previously described [85]. Immunofluorescence was detected by fluorescence microscopy (Olympus).

### Mouse Injections, Necropsy, Histopathology

The ability to form tumors and metastasize was analyzed by injecting cells with repressed Bmi-1 into nude mice. Mice were bred and maintained under SPF conditions in the Department of Animal Center, Cancer Center, Sun Yat-Sen University, as approved by the China Care Committee Institute. Ten healthy female nude mice, which were four- to six- weeks old, were randomly assigned to each group. Each mouse was injected in the fat pad with 2 × 10^6 ^cells in PBS solution. Tumor growth was measured by caliper, and tumor volume was calculated according to the formula: length × width^2 ^× 0.52, as described previously [86]. All mice were sacrificed on the sixth week after injection. The primary tumor and lung tissues of each mouse were removed, weighed and embedded in 10% paraffin. Each tissue was chopped into small pieces. Total protein was extracted to detect Bmi-1 expression from the primary xenografts. Each section from the primary xenografts and lung tissues was subjected to H&E staining, according to standard protocols, for histological examination and metastasis evaluation. The nodes of lung metastasis were quantified by counting metastatic lesions in ten sections (10 μm per section in a series). Data were collected by counting the total numbers of metastatic lesions from ten sections. Sections of primary tumors and lung lesions were used to detect the expression of the markers (Bmi-1, β-catenin, fibronectin) by IHC, as described previously.

### Statistical analysis

The Chi-Square test was employed to evaluate the differences in Bmi-1 expression between the two categories of tissues. For assessment of the correlation between clinical features and Bmi-1 expression in breast cancer, *P *values were calculated by using the Chi-Square test or the Fisher's exact test. Relative risks (RRs) of death associated with Bmi-1 expression and other predictive variables were estimated by using the univariate and multivariate Cox proportional hazards model. The overall survival curve was plotted using Kaplan-Meier survival analysis and compared by the log-rank test. Result variations for the chamber invasion assays, wound healing assay, soft agar assay, tumor volume, tumor weight and lung metastasis lesions in mice, described as mean ± SD, were assessed using the two-tailed Student's *t *test. A value of *P *< 0.05 was considered significant (two tailed) by using SPSS 16.0.

## Abbreviations used

Bmi-1: B-lymphoma Moloney murine leukemia virus insertion region-1; EMT: epithelial-mesenchymal transition; HER-2/neu: Human Epidermal Growth Factor Receptor 2; NPCs: nasopharyngeal cancer cells; NPECs: nasopharyngeal epithelial cells.

## Competing interests

The authors declare that they have no competing interests.

## Authors' contributions

BHG and YF were involved in the design, performed the experiments, and drafted the manuscript. RZ, LHX, MZL participated in sample collection. HFK and LBS reviewed the manuscript. MSZ conceived the idea for the study, contributed to the overall experiment design and revised the manuscript. All authors read and approved the final manuscript.

## Supplementary Material

Additional file 1 **Table S1(PDF). Distribution of patient characteristics by survival status**. Click here for file

Additional file 2 **Table S2(PDF). The patients' information in different studies**. Click here for file

Additional file 3 **Table S3(PDF). Antigen retrieval method, recourse of antibody and developmental time in different studies**. Click here for file

Additional file 4 **Table S4(PDF). Positive criteria used in different studies**. Click here for file

Additional file 5 **Table S5(PDF). The description of results in different studies**. Click here for file
