# Supplementary material for: Bmi-1 promotes invasion and metastasis, and its elevated expression is correlated with an advanced stage of breast cancer
Source: Mol Cancer. 2011 Jan 28;10:10. doi: 10.1186/1476-4598-10-10 (PMC3038148; doi:10.1186/1476-4598-10-10)
Supplement: Additional file 1 — Table S1(PDF). Distribution of patient characteristics by survival status. [file 1476-4598-10-10-S1.PDF]

Supplementary Table 1. Distribution of patient characteristics by survival status

	Survival status				χ^2	*P*-value
	Alive		Dead			
	N	(%)	N	(%)		
Age					0.531	*0.466*
≤45 year	63	(64.3)	35	(35.7)		
>45 year	97	(68.8)	44	(31.2)		
T Classification					22.070	*<0.001*
T1	36	(81.8)	8	(18.2)		
T2	93	(71.5)	37	(28.5)		
T3	26	(56.5)	20	(43.5)		
T4	5	(26.3)	14	(73.7)		
N Classification					25.791	*<0.001*
N0	71	(83.5)	14	(16.5)		
N1	80	(62.5)	48	(37.5)		
N2	6	(28.6)	15	(71.4)		
N3	3	(60.0)	2	(40.0)		
M Classification					19.531	*<0.001*
M0	143	(73.3)	52	(26.7)		
M1	17	(38.6)	27	(61.4)		
Clinical Stage					28.705	*<0.001*
Ⅰ	21	(95.5)	1	(4.5)		
Ⅱ	68	(77.3)	20	(22.7)		
Ⅲ	54	(63.5)	31	(36.5)		
Ⅳ	17	(38.6)	27	(61.4)		
ER Presence					1.208	*0.272*
Negative	75	(63.6)	43	(36.4)		
Positive	85	(70.2)	36	(29.8)		
PR Presence					9.790	*0.002*
Negative	59	(56.2)	46	(43.8)		
Positive	101	(75.4)	33	(24.6)		
HER-2 Presence					0.692	*0.406*
Negative	36	(76.6)	11	(23.4)		
Positive	70	(70.0)	30	(30.0)		
Bmi-1 Expression					13.827	*<0.001*
Low	57	(85.1)	10	(14.9)		
High	103	(59.9)	69	(40.1)		
