# Supplementary material for: Bmi-1 promotes invasion and metastasis, and its elevated expression is correlated with an advanced stage of breast cancer
Source: Mol Cancer. 2011 Jan 28;10:10. doi: 10.1186/1476-4598-10-10 (PMC3038148; doi:10.1186/1476-4598-10-10)
Supplement: Additional file 2 — Table S2(PDF). The patients' information in different studies. [file 1476-4598-10-10-S2.PDF]

Supplementary Table 2. The patients' information in different studies

Author	Number	Diagnosis	Population	Mean Age	Follow-up	Method	TNM (%, N)
Nalwoga H	192	1990-2002	African (Ugandan)	46.2Y (18-80)	9 M(0.5-108)	TMA and IHC	2003 (WHO)<br>Stage 1-2: 9% (2)<br>Stage 3: 36% (8)<br>Stage 4: 54% (12)
Pietersen AM	295	1984-1995	Dutch	Unknown	10.2 Y	TMA and IHC	Stage 1-2: 295 samples
Choi YJ	960	1995-2002	Korean	47Y (20-80)	77.1 M(8-149)	TMA and IHC	6th AJCC<br>Stage Ⅰ: 20.2% (94)<br>Stage Ⅱ: 55.6% (534)<br>Stage Ⅲ: 24.2% (232)
Kim JH	71	2000-2002	Korean	Unknown	Unknown	IHC	TNM residual tumor classification
Arners JB	176	1996-2001	Norwegian	60Y (49-72)	69M (10-105)	TMA and IHC	Unknown
Guo BH	252	1999-2001	Chinese	47Y (26-78)	59M (4-78)	IHC	2002 (UICC)<br>Stage Ⅰ: 9.1% (23)<br>Stage Ⅱ: 37.3% (94)<br>Stage Ⅲ: 35.7% (90)<br>Stage Ⅳ: 17.9% (45)
