# Supplementary material for: Bmi-1 promotes invasion and metastasis, and its elevated expression is correlated with an advanced stage of breast cancer
Source: Mol Cancer. 2011 Jan 28;10:10. doi: 10.1186/1476-4598-10-10 (PMC3038148; doi:10.1186/1476-4598-10-10)
Supplement: Additional file 3 — Table S3(PDF). Antigen retrieval method, recourse of antibody and developmental time in different studies. [file 1476-4598-10-10-S3.PDF]

Supplementary Table 3. Antigen retrieval method, recourse of antibody and developmental time in different studies

Author	Antigen Retrieval Method	Antibody	Development
Nalwoga H	Tris–EDTA pH 9.0 microwaving (750 W for 10 min) (350 W for 20 min)	6C9 60 min, RT	10 min
Pietersen AM	citrate buffer, Unknown	Millipore, F6, Anti-mouse 1:400	Unknown
Choi YJ	citrate buffer, microwaving	Upstate Biotechnology Lake Placid, NY, USA	5 min
Kim JH	citrate buffer (pH=6.0), microwaving	Upstate Biotechnology, Lake Placid, NY, USA Anti-mouse 1:100, 90min, RT	Unknown
Arners JB	Tris–EDTA pH 9.0, microwaving	Upstate Biotechnology, Billerica, MA, USA Clone F6, 1:800, overnight at 4°C	
Guo BH	EDTA pH 8.0, microwaving	Upstate Biotechnology, Lake Placid, USA clone F6, 1:100, overnight at 4°C	10 min
