# Supplementary material for: Bmi-1 promotes invasion and metastasis, and its elevated expression is correlated with an advanced stage of breast cancer
Source: Mol Cancer. 2011 Jan 28;10:10. doi: 10.1186/1476-4598-10-10 (PMC3038148; doi:10.1186/1476-4598-10-10)
Supplement: Additional file 4 — Table S4(PDF). Positive criteria used in different studies. [file 1476-4598-10-10-S4.PDF]

Supplementary Table 4. Positive criteria used in different studies

Author	Evaluation
Nalwoga H	A staining index (values 0–9) was determined by multiplying the score for intensity of staining(none=0, weak=1, moderate=2 and strong=3) with the score for proportion of tumor cells stained (<10%=1,10–50%=2, >50%=3).<br>The majority of cases (75%) had staining index 0, and therefore the cut off was 0=negative and 1–9=positive.
Pietersen AM	38% patients having high levels of expression
Choi YJ	The staining intensity was subclassified as 0 (negative), 1 (weak), 2 (moderate), and 3 (strong). Positive cells were quantified as a percentage of the total number of tumor cells with observation of 100 cells in 5 high power field (9400), and assigned to one of five categories: 0:<5%, 1: 5–25%, 2: 26–50%, 3: 51–75% and 4:>75%. The score of percentage of positive tumor cells, and the score of staining intensity were added to generate the immunoreactive score (IS) for each tumor specimen. The cases were grouped by IS value as 0, 1+ (IS 1.2), 2+ (IS 3.4), 3+ (IS 5.6) and 4+ (IS 7).<br>The slide of higher than 2+ was classified as a positive case.
Kim JH	Moderate to strong nuclear staining was regarded as a positive reaction. The distribution of Bmi-1 was scored on a semi-quantitative scale, as follows: negative (<10% of tumor positive), focally positive(10–50% of tumor positive), and diffusely positive (>50% of tumor positive).
Arners JB	The intensity of reactivity was graded 0, (no reactivity) to 3 (strong reactivity). Tumor cell area was graded 1(0–10%), 2 (11–50%) and 3 (>50%). A composite staining index (SI) was defined as the product of the intensity and area scores, giving values from 0 to 9. Expression categories were initially based on quartiles for SI in the present dataset, considering also the distribution plots and the number of cases and events when dichotomous variables were constructed. After evaluation, the SI median (SI=3) was chosen for analysis of associations and outcome.

Guo BH	Positive cells were quantified as a percentage of the total number of tumor cells in 10 low power field (40X) and assigned to one of four categories: ≤5% of the cells (1 point), 6% to 35% of the cells (2 points), 36% to 70% of the cells (3 points), ≥71% of the cells (4 points). The staining intensity was subclassified as negative staining (1 point), weak staining (2 points), moderate staining (3 points) and strong staining (4 points). A final score was then calculated by multiplying the above two scores. If the final score was >4, Bmi-1 expression was considered high, otherwise, Bmi-1 expression was considered negative.

AJCC: the American Joint Committee on Cancer

UICC: International Union Against Cancer
