# Supplementary material for: Bmi-1 promotes invasion and metastasis, and its elevated expression is correlated with an advanced stage of breast cancer
Source: Mol Cancer. 2011 Jan 28;10:10. doi: 10.1186/1476-4598-10-10 (PMC3038148; doi:10.1186/1476-4598-10-10)
Supplement: Additional file 5 — Table S5(PDF). The description of results in different studies. [file 1476-4598-10-10-S5.PDF]

Supplementary Table 5. The description of results in different studies

Author	Results
Nalwoga H	46 tumors (25%) were positive for Bmi-1 staining. Bmi-1 positivity was mostly associated with low histological grade, low mitotic counts and ER positivity. Bmi-1 expression was inversely associated with the TNP. No survival Analysis
Pietersen AM	High expression of Bmi-1 predicted better OS. The 'protective' effect of Bmi-1 expression was limited to lymph node-positive patients. Bmi-1 was correlated low grade and positive ER. Bmi-1 was an independent prognostic factor
Choi YJ	511 (53.2%) cases had positive staining. Bmi-1 expression was associated with smaller tumor size, negative axillary lymph node, earlier stage, low nuclear grade, positive ER and positive PR. Bmi-1 was an independent prognostic factor for OS.
Kim JH	58% (30 in 44) was observed more intensely in the invading fronts than in the central portions of the primary invasive breast cancers. Bmi-1 oncoprotein was negative, focally and diffusely positive in 27 (38%), 21 (30%), and 23 (32%) cases. There was a positive correlation between Bmi-1 expression and axillary LN metastases or ER.
Arners JB	75 (42.6%) cases had positive staining. There were positive associations between Bmi-1 with ER and PR positivity, negative correlation between Bmi-1 and basal like profiles. Univariate survival analysis did not show a significant influence of Bmi-1 status on overall survival.
Guo BH	72.2% (182) of cases were defined as high expression. 96.5% (241) cases showed positive staining. Bmi-1 expression was strongly correlated with large tumor size, lymph node involvement, distant metastasis, and advanced clinical stage. High Bmi-1 expression was associated with an unfavorable prognosis.
